# Non-Targeted Analysis Using Gas Chromatography-Mass Spectrometry for Evaluation of Chemical Composition of E-Vapor Products

**DOI:** 10.3389/fchem.2021.742854

**Published:** 2021-09-29

**Authors:** Niti H. Shah, Michael R. Noe, Kimberly A. Agnew-Heard, Yezdi B. Pithawalla, William P. Gardner, Saibal Chakraborty, Nicholas McCutcheon, Hannah Grisevich, Thomas J. Hurst, Michael J. Morton, Matt S. Melvin, John H. Miller IV

**Affiliations:** Center for Research and Technology, Altria Client Services LLC, Richmond, VA, United States

**Keywords:** non-targeted analysis, electronic vapor products, ENDS, aerosol, e-liquids, semi-quantitative analysis, GC-MS, e-cigarettes

## Abstract

The Premarket Tobacco Product Applications (PMTA) guidance issued by the Food and Drug Administration for electronic nicotine delivery systems (ENDSs) recommends that in addition to reporting harmful and potentially harmful constituents (HPHCs), manufacturers should evaluate these products for other chemicals that could form during use and over time. Although e-vapor product aerosols are considerably less complex than mainstream smoke from cigarettes and heated tobacco product (HTP) aerosols, there are challenges with performing a comprehensive chemical characterization. Some of these challenges include the complexity of the e-liquid chemical compositions, the variety of flavors used, and the aerosol collection efficiency of volatile and semi-volatile compounds generated from aerosols. In this study, a non-targeted analysis method was developed using gas chromatography-mass spectrometry (GC-MS) that allows evaluation of volatile and semi-volatile compounds in e-liquids and aerosols of e-vapor products. The method employed an automated data analysis workflow using Agilent MassHunter Unknowns Analysis software for mass spectral deconvolution, peak detection, and library searching and reporting. The automated process ensured data integrity and consistency of compound identification with >99% of known compounds being identified using an in-house custom mass spectral library. The custom library was created to aid in compound identifications and includes over 1,100 unique mass spectral entries, of which 600 have been confirmed from reference standard comparisons. The method validation included accuracy, precision, repeatability, limit of detection (LOD), and selectivity. The validation also demonstrated that this semi-quantitative method provides estimated concentrations with an accuracy ranging between 0.5- and 2.0-fold as compared to the actual values. The LOD threshold of 0.7 ppm was established based on instrument sensitivity and accuracy of the compounds identified. To demonstrate the application of this method, we share results from the comprehensive chemical profile of e-liquids and aerosols collected from a marketed e-vapor product. Applying the data processing workflow developed here, 46 compounds were detected in the e-liquid formulation and 55 compounds in the aerosol sample. More than 50% of compounds reported have been confirmed with reference standards. The profiling approach described in this publication is applicable to evaluating volatile and semi-volatile compounds in e-vapor products.

## Introduction

Electronic nicotine delivery system (ENDS) product usage has increased in popularity over the past decade as a potential alternative to combustible cigarettes for the adult tobacco consumer (Ayers et al., 2011; Adkison et al., 2013; Delnevo et al., 2016), and usage continues to increase (McMillen et al., 2015; Rigotti et al., 2015; Rawlinson et al., 2017). ENDSs are non-combustible tobacco products and are also referred to as electronic cigarettes (e-cigs), vapes, vaporizers, vape pens, or e-vapor products. Their designs have evolved from the first-generation devices (“cig-a-likes”) to devices with disposable, prefilled cartridges or “pods” and “mods” with user-controllable settings, such as wattage, voltage, and temperature control (Zhu et al., 2014; Talih et al., 2017; FDA, 2019c). ENDS products aerosolize the e-liquid that is typically composed of a mixture containing propylene glycol (PG), vegetable glycerol (VG), nicotine, and flavors (Hahn et al., 2014; Flora et al., 2016; FDA, 2019c; Cunningham et al., 2020).

In June 2019, the Food and Drug Administration (FDA) provided final guidance to the industry for submitting a premarket tobacco product application (PMTA) for ENDS products (FDA, 2019b). This guidance recommended that all ENDS products, including e-liquids and devices, be evaluated in order to ensure that these new products would be appropriate for the protection of public health. These recommendations included the evaluation of both chemical and physical characterization of the product and product performance across the lifespan of the device under both intense and non-intense use conditions (FDA, 2019b). The guidance also recommends the characterization of these product attributes for inclusion in stability studies used for determining product shelf-life. For chemical characterization, the guidance recommends reporting a specific list of harmful and potentially harmful constituents (HPHCs), as well as other constituents of toxicological concern, contained in the product or delivered by the product (FDA, 2019b). The list of 33 HPHCs includes combustion-related compounds (Wagner et al., 2018), thermal degradation products from humectants and other compounds specific to the product category such as flavorants, and potential impurities from raw materials (Flora et al., 2016; Uchiyama et al., 2020; Li et al., 2021). Due to the significantly low temperatures (i.e., < 350°C) typically used to generate the aerosol within ENDS products, the combustion (∼900 °C)-related HPHCs formed by conventional cigarettes are not typically formed or are produced at significantly lower levels in aerosols of ENDS products compared to cigarette smoke (Goniewicz et al., 2014; Tayyarah and Long, 2014; Margham et al., 2016; Wagner et al., 2018). Thermal degradation of the e-liquid has been reported to occur at temperatures typically required for the aerosol formation process (Flora et al., 2017; Uchiyama et al., 2020; Li et al., 2021). Other compounds specific to the product category are observed in the aerosol through direct transfer from the e-liquid to the aerosol. In order to accurately quantitate these HPHCs, analytical methods that target the specific analytes are typically developed and validated according to International Council of Harmonization (ICH) guidelines to generate data that are used for regulatory reporting. The characterization of ENDS products for constituents of toxicological concern contained in the product or delivered by the product may require an additional type of analysis to complement the targeted analysis for HPHCs as described above. This type of analysis requires performing chemical characterization that is non-selective and provides the detection of constituents across a wide range of chemical classes, often referred to as non-targeted analysis (NTA).

NTA techniques are widely used in the environmental, food, and plastic industries and for the evaluation of biological samples to identify impurities, contaminates, or compounds of concern (Andra et al., 2017; Keppler et al., 2018; Sobus et al., 2018; Martínez-Bueno et al., 2019). Coupling chromatographic separation with mass spectrometry detection and custom internal mass spectral libraries improves the peak identification of compounds within these mixtures (Rawlinson et al., 2017). Current analytical methods used for NTA span a broad range of techniques, including unit-mass resolution gas chromatography-mass spectrometry (GC-MS), two-dimensional gas chromatography time-of-flight mass spectrometry (GCxGC-TOF MS), ultra-high-resolution Fourier transform ion cyclotron resonance mass spectrometry, and liquid chromatography coupled with high-resolution Orbitrap or time-of-flight mass spectrometry (LC-MS) (Reichenbach et al., 2012; Herrington and Myers, 2015; Andra et al., 2017; Junot and Witting, 2017; Sobus et al., 2018; Knorr et al., 2019; Martínez-Bueno et al., 2019; Savareear et al., 2019). The data obtained from the analysis are utilized to determine the chemical structure of the detected compounds. Some NTA methods can provide semi-quantitative concentrations of all compounds detected in the analysis. Compound structural identification and semi-quantitative concentration provided by NTA for each compound can then be used by toxicologists to perform risk assessments.

To fully characterize e-vapor products, additional screening methods capable of evaluating the chemical composition of e-liquids and aerosols must be developed. NTA screening methods can prove to be useful tools for the evaluation of e-vapor products for the presence of compounds that may potentially be of toxicological concern in addition to HPHCs. There are some NTA methods reported in the literature for characterization of e-vapor products (Reichenbach et al., 2012; Herrington and Myers, 2015; Rawlinson et al., 2017). Herrington and Myers described non-targeted GC-MS analysis of e-liquids where the sample was collected manually on a thermal desorption tube prior to desorption and qualitative analysis on a quadrupole mass analyzer. Their analysis resulted in detectable levels of more than 115 volatile organic compounds (VOCs) and semi-volatile organic compounds in one 40 ml puff. Several compounds, including formaldehyde, acrolein, acetaldehyde, and siloxanes, were detected in the e-vapor aerosol and not in the original e-liquid, which suggested that these compounds were formed during aerosolization (Herrington and Myers, 2015). The sensitivity of their method was impacted for several analytes due to overloading of PG and VG in the system. Rawlinson et al. (2017) described a non-targeted method for e-vapor products using thermal desorption gas chromatography time-of-flight mass spectrometry with a 5 ng/puff limit of detection (LOD). The method employed a heart-cutting process with a Deans Switch to avoid saturation of the mass analyzer by high-abundance ingredients (e.g., PG, VG, and nicotine). However, this process eliminates the identification of compounds that co-elute with these high-concentration analytes. The LOD for their method was established based on a toxicologically relevant threshold and the ability to identify compounds. The method by Rawlinson et al. (2017) was generally compatible for analysis of volatile organic and nitrogen-containing compounds but was not applicable for identification of very low-molecular-mass compounds, some organic acids, and high-boiling-point compounds. The method did employ an automated workflow that increased data throughput significantly; however, it was challenged by partial deconvolution of some chromatographic peaks due to low abundance and co-elution of multiple compounds within one peak. Chemical identification is a major challenge with NTA due to the composition of the matrix, including flavor ingredients, and high-abundance ingredients present in the e-liquid’s carrier system. Additional identification challenges arise particularly for unknowns due to insufficient compound libraries, mass spectral ions that match with different chemical structures within the mixture, and co-elution of multiple compounds due to complexity of the matrices (Reichenbach et al., 2012). In addition, the National Institute of Standards and Technology (NIST) mass spectral library is largely based upon known compounds analyzed with unit-mass-resolution GC-MS instruments, and the library in select cases may include multiple mass spectra associated with a single chemical compound. A comprehensive chemical characterization of the aerosol generated by a heated tobacco product (HTP) and mainstream smoke from a reference cigarette was reported using two-dimensional gas chromatography coupled to time-of-flight mass spectrometry (GCxGC-TOFMS) and liquid chromatography coupled to high-resolution accurate mass spectrometry (LC-HRAM-MS) (Knorr et al., 2019; Arndt et al., 2020; Bentley et al., 2020). This comprehensive non-targeted analysis workflow used two parallel platforms utilizing multiple analytical methods to maximize the chemical space coverage in order to fully characterize mainstream cigarette smoke and heated tobacco aerosols. Although this technique was required for characterization of these products, due to the highly complex matrix, the instrumentation is expensive and requires specialized expertise to operate. In addition, two-dimensional GC is not required for the e-vapor matrix since it is relatively less complex and has been reported to contain an order of magnitude fewer compounds than HTPs (Rawlinson et al., 2017).

Here, we describe an approach to non-targeted analysis using unit-mass-resolution GC-MS with the electron impact ionization mode (EI) and a new automated data analysis process workflow. The method uses relatively inexpensive GC-MS instruments and software, making it a practical method for many analytical testing laboratories. The Agilent MassHunter Unknowns Analysis software assisted in peak detection and deconvolution, followed by identification of compounds using the NIST library and a custom in-house library. A traditional validation of an e-vapor NTA method was not possible due to the absence of a standard method or guidance document. Because of this and unique challenges associated with any non-targeted analysis, it was necessary to modify validation experiments to demonstrate that our method was fit for its intended purpose. Most validation experiments were modified to include the analysis of known compounds for fortification, which included degradation products from nicotine, impurities from PG/VG, and flavor-related compounds. Since the fortification compounds were prepared and added to the blank matrices at known concentrations, comparisons could be made between actual and estimated concentrations. Critical validation parameters (i.e., accuracy, precision, selectivity, and LOD) were established to demonstrate that the method is appropriate for analysis of e-liquids and aerosols of e-vapor products. The modified experiments conducted for method validation in this article could aid in defining best practices leading to standardized guidance for the validation of semi-quantitative NTA methods. The technique described here was used for stability evaluation of a commercial e-vapor product using differential analysis to determine new compounds that formed between an initial assessment (T = 0) and after 6 months (T = 6). The method and complete workflow were integrated to an internal Laboratory Information Management System (LIMS) and was accredited under the ISO 17025 scope of accreditation.

## Materials and Methods

### Overall Non-Targeted Analysis Workflow

**FIGURE 1:**
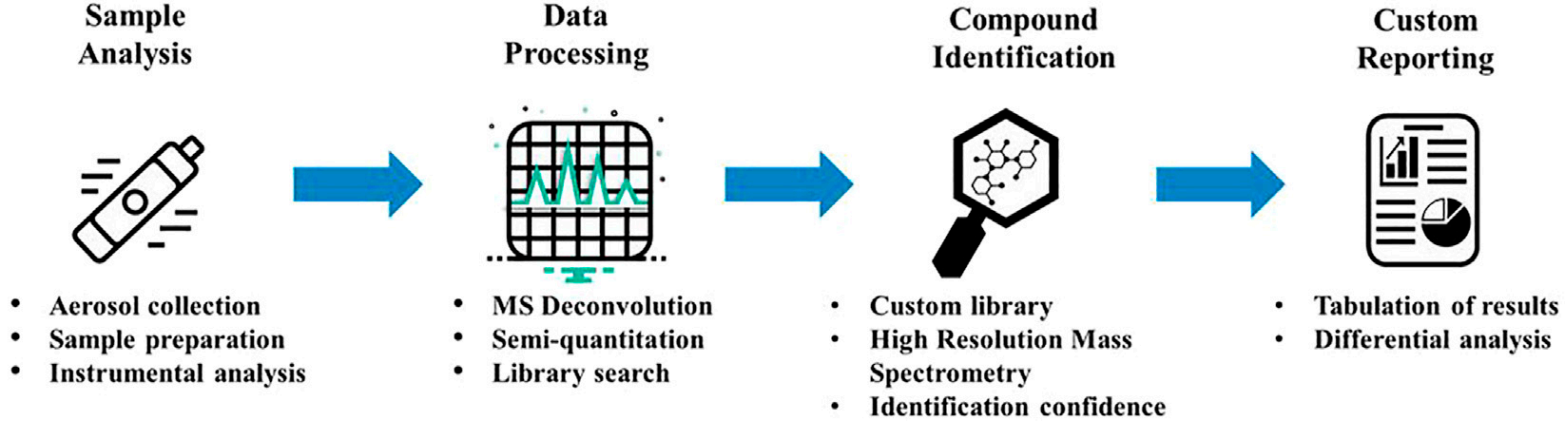
Workflow for NTA analysis by GC-MS.

We developed an NTA workflow that includes four major steps: sample analysis, data processing, compound identification, and custom reporting. Illustrated in Figure 1 is an overview of each step of the workflow. Sample analysis and data processing were two steps in the workflow that required optimization of parameters to ensure that the method was sensitive, selective, and reproducible. An automated data processing approach was developed and employed to minimize the subjectivity and time needed for data processing and review. Our NTA workflow leverages MassHunter Unknowns Analysis, which is part of the Agilent MassHunter Quantitative software package (Agilent Technologies, Santa Clara, CA), and was specifically designed for non-targeted screening and incorporates mass spectral deconvolution, compound identification, and quantitation capabilities. This data processing software was a critical step to the workflow, and development of the processing method required optimization of processing parameters to ensure that repeated analyses provide reproducible compound identification and quantitation in samples. The peak deconvolution algorithm extracts ions from background noise and reconstructs spectra of the individual components from retention time and peak shape information. Compound identification was achieved using both the NIST mass spectral library and a custom in-house library that contains compounds that have been confirmed with reference standards. Using this custom library allowed us to track compounds based on retention times and relative retention times that resulted in improved consistency of compound identifications.

Unknown identification can be extremely challenging and may require highly experienced subject matter experts and tools for compound identification and structural characterization [e.g., high-resolution mass spectrometry (HRMS) and nuclear magnetic resonance (NMR)]. In instances where compounds could not be identified based on our custom library or the NIST library, a secondary analysis was required. The secondary analysis was performed using a Thermo Scientific GC-Orbitrap system that provides high-resolution accurate mass spectra to aid in the identification of unknown compounds. Using the GC-Orbitrap in both electron ionization (EI) and chemical ionization (CI) modes allowed for the determination of the molecular formula from the molecular ion, base peak, and other peak fragments for mass spectral interpretation and structural elucidation. Based on the tentative chemical structure identification, unknowns were subsequently confirmed by custom synthesis or acquiring reference standards. Once the data were processed and compounds had been identified, the results were imported into the laboratory information management system (LIMS) data application to ensure data integrity and reporting. The results were then exported from LIMS into the Tableau software application (Tableau Software, Seattle, WA) for final reporting. Tableau was used to create the custom report templates and perform blank subtractions using specified criteria. The software was also used for tracking individual compounds over multiple time points to evaluate any trends and changes in the composition of the e-liquid or aerosol from a product over time.

### Sample Generation

**FIGURE 2:**
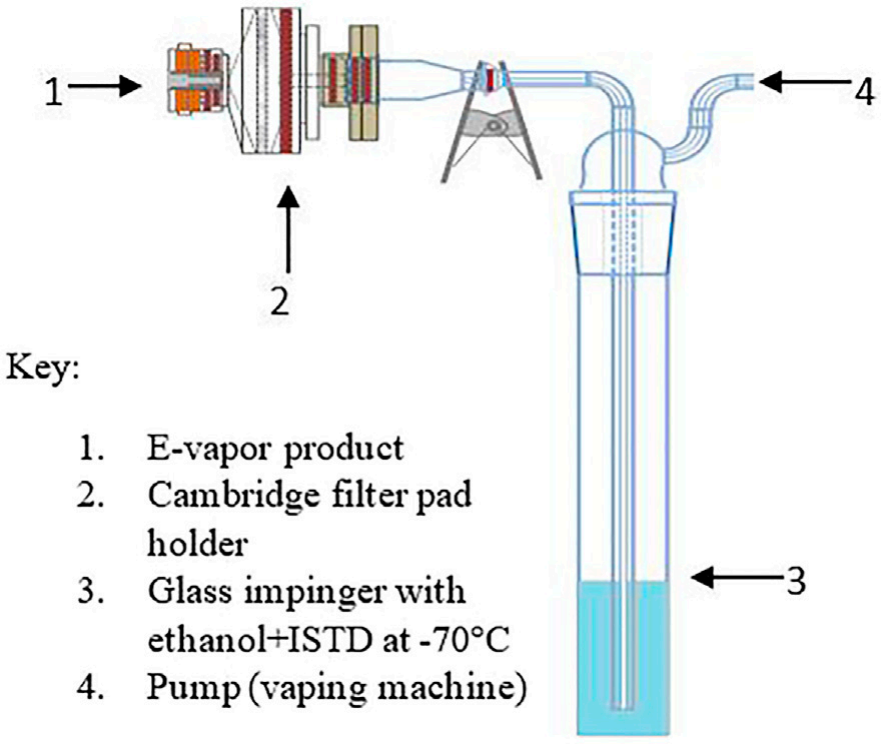
Schematic for aerosol collection.

The aerosol was collected on a linear aerosol collection machine (Borgwaldt LX20; Hamburg, Germany) that was exclusively used for e-vapor collection to avoid any potential contamination from prior use with conventional cigarette products. The e-vapor aerosols were collected on a 55 mm Cambridge filter pad (CFP) with a trailing impinger containing 10 ml of the extraction solvent [absolute ethanol containing 10 μg/ml of the internal standard (ISTD) 6-methyl coumarin] chilled at −70°C using a dry ice/isopropanol slurry (see Figure 2). The puffing regime parameters consisted of a 55 ml puff volume, a 5 s puff duration, a 30 s puff interval, and a square wave puff profile. A total of 140 puffs were collected. The aerosol trapping efficiency was evaluated prior to the final collection process. The trapping efficiency experiment results indicated that the aerosol sample collection using the Cambridge filter pad attached to a single trailing impinger was acceptable for the machine smoking regime used.

Once the designated number of puffs was collected, the pad and extraction solvent from the impinger were both combined in a 20 ml glass vial, followed by mixing for 30–60 min on an inversion-type rotator (∼15 rpm). After the extraction was complete, an aliquot of the sample was transferred to an autosampler vial and analyzed by GC-MS.

The corresponding e-liquid(s) used to generate the aerosol using the above-described methodology was also analyzed according to the procedure outlined below. The target extraction weight of each batch of e-liquid samples was determined from the average aerosol mass collected for all replicates of the same product analyzed for aerosols. E-liquid samples were generated by removing the e-liquid from a product cartridge or from the bulk formulation container. To collect the e-liquid from a product, e-vapor sample cartridges were centrifuged for 2–6 min at 1,000–6,000 rpm. The e-liquid (∼0.600–0.800 ± 0.050 g as determined based on the aerosol mass for the corresponding e-vapor product analyzed) was weighed into a 20 ml amber screw cap vial and combined with 10 ml of the extraction solvent containing the internal standard. The samples were then mixed on an inversion-type rotator (∼15 rpm) for 30–60 min. The last steps were to transfer an aliquot into an autosampler vial and analyze by GC-MS.

### Gas chromatography-Mass Spectrometry Method Conditions

**FIGURE 3:**
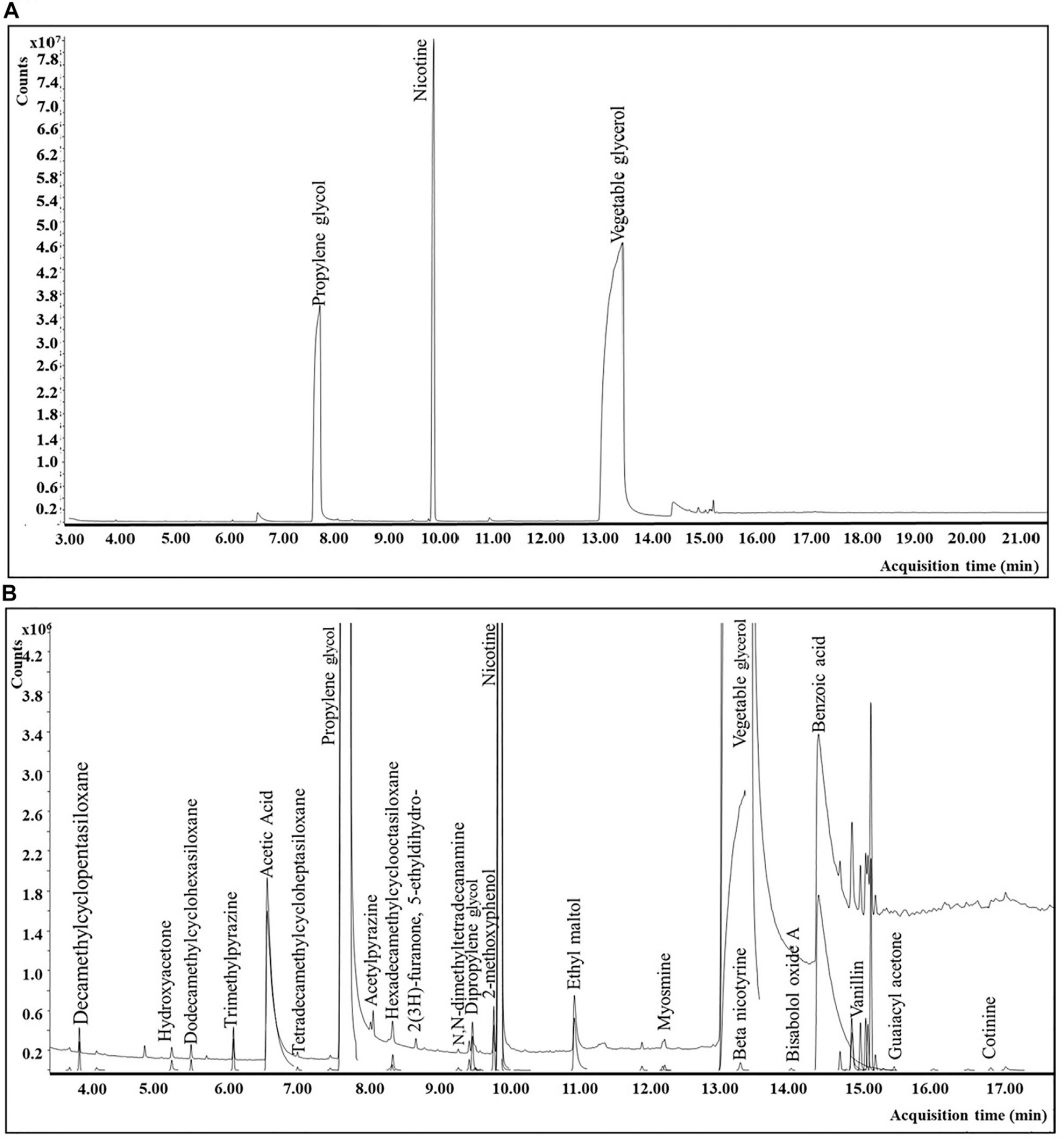
GC-MS chromatograms for a commercial e-vapor product: (**A**) total ion chromatogram (TIC) for the e-vapor aerosol sample. (**B**) Magnified version (approximately ×10) of Figure 3A including peak deconvolution.

Aerosol and e-liquid samples were analyzed on an Agilent technologies (Santa Clara, CA) single quadrupole GC-MS system (7890 B with 5977 A) equipped with an EI source at 230°C, a quadrupole mass spectrometer at 150°C, and the transfer line temperature at 260°C. Mass spectra were recorded in the full scan mode with a mass range of 35–450 amu and 3.5 scan/sec. The instrument was operated in constant flow at 1.2 ml/min with an inlet temperature of 260°C and an injection volume of 1 µl (split 5:1). Chromatographic separation was achieved using a Restek Stabilwax^®^ (Restek Corporation; Bellefonte, PA) GC column (30 m × 0.25 mm × 0.25 μm) with an infused 5 m Restek Integra guard column. The GC oven was initially held at 60°C for 1.25 min, followed by a 15°C/min ramp to 210°C with a 2 min hold time, followed by a 30°C/min ramp to 260°C with a 9 min hold time. The total run time was 23.92 min. A typical total ion chromatogram for the e-vapor aerosol extract using the sample preparation and GC-MS method conditions described above is represented in Figure 3.

### Data Processing and Reporting

The instrument raw data files were processed using a data analysis workflow through Agilent MassHunter Unknowns Analysis software version B.08.00 (Agilent Technologies; Santa Clara, CA). The automated data processing workflow included automatic peak detection, deconvolution, library searches for compound identifications, and calculation of the estimated concentrations. The method used for this data processing contained predetermined parameters and thresholds, such as signal-to-noise ratio (S/N), peak detection limits, deconvolution, library search criteria, and compound identification and target match criteria. These parameters were optimized during method development and represent a balance between the software’s ability to correctly identify compounds and the maximum number of peaks detected while maintaining acceptable mass spectral quality. More details on the MassHunter Unknowns Analysis parameters used for the data processing method can be found in the Supplementary Material. Semi-quantitation of the detected compounds was achieved using a manual response factor that was calculated from an analog internal standard. The manual response factor was calculated using the peak area of the internal standard (ISTD) and included the extraction volume and average aerosol masses of the samples analyzed as described by following equation:

$$Manual\;Response\;Factor\;=\;\frac{\left(ISTD\;Area/ISTD\;Conc\;\right)*\;\left(Sample\;Wt.\right)}{Volume},$$

where, ISTD Area is the base peak area for the internal standard 6-methylcoumarin, ISTD Conc is the known concentration of ISTD (µg/ml), sample Wt. refers to the weight of the e-liquid or aerosol mass (mg), and volume refers to the extraction solution volume used for the extraction of each sample (ml). The calculated response factor was entered into the data processing method to determine the estimated concentration for each analyte. During data processing, compound identifications for peaks in the study samples were obtained by comparing the mass spectra from the samples to the NIST 2017 mass spectral library in addition to the in-house custom mass spectral library. The in-house custom library contained known peak identifications with their corresponding peak retention times and mass spectra. Agilent MassHunter Unknowns Analysis software compared the mass spectra and retention times of the library compounds to those in the samples, resulting in a tentative identification based on criteria specified by method parameters.

Compound identifications were separated into five classifications (i.e., confirmed, high, medium, low, and NA) based on identification confidence and the NIST MS library match factor score (Table 1). Confirmed, high, medium, and low identification confidence classifications were based on mass spectrum match factor scores from an Automated Mass spectral Deconvolution and Identification System (AMDIS; NIST, Gaithersburg, MD), a secondary deconvolution software available with the NIST MS library that was used to investigate or confirm peak identifications and to assign identification confidence levels. It was observed that a high match factor score did not always represent an acceptable mass spectral library match. Identification confidence classification assigned to every chemical component was determined by visual inspection of the mass spectrum by an experienced analyst, in addition to the match factor score. Compounds labeled with a confirmed identification confidence classification were positively identified by comparing the compound’s mass spectra and relative retention time (RRT) to a reference standard. Compounds that did not have an acceptable mass spectral library match (i.e., match factors lower than 500) were classified as unknowns (NA).

**TABLE 1:** Peak identification confidence criteria.

Identification confidence	NIST MS match factor score criteria
Confirmed	Identification confirmed by comparison of the compound mass spectrum and relative retention time (RRT), to a reference standard
High	850–1,000
Medium	700–849
Low	500–699
NA	Unknown compound

### Chemicals

The following compounds were purchased from Sigma-Aldrich (St. Louis, MO): piperonal (99.0%), 2,3,5-trimethylpyrazine (99.0%), menthone (99.0%), (E)-beta-damascone (99.0%), cinnamic acid methyl ester (99.0%), myosmine (99.0%), cotinine (98.5%), and the internal standard (ISTD) 6-methyl coumarin (99.0%). Hydroxyacetone (96.0%) was purchased from Santacruz Biotechnology (Dallas, TX). 200 proof ethanol was purchased from Pharmco-Aaper. Propylene glycol (PG) and vegetable glycerol (VG) were purchased from Spectrum (New Brunswick, NJ).

### Test Samples for Method Validation

For method validation, a mixture of eight compounds (validation fortification standards)—hydroxyacetone, piperonal, 2,3,5-trimethylpyrazine, menthone, (E)-beta-damascone, cinnamic acid methyl ester, myosmine, and cotinine—were selected by classification (e.g., flavors, degradation products, etc.) and retention time to ensure that the separation of compounds was well distributed across the entire chromatographic method run time. All matrices used for the method validation experiments were fortified using the eight compounds. Test samples (F1–F5) for method assessment listed in Table 2 were prepared with a mixture of nicotine, water, PG, and VG to cover the wide range of PG/VG concentrations available in commercial e-vapor products. The samples contained 0% or 2.5% nicotine by weight (NBW) and 0% or 15% water. PG/VG is the ratio of the percent remainder, minus the sum of % H_2_O and % NBW: [(100-(%H_2_O+ %NBW)]. The ratio of PG/VG is based on the amount remaining such that %PG+%VG = 100%. (see Table 2). In addition to the method assessment samples, the e-liquid and aerosol of two e-vapor product prototypes aged to approximately 2 years under controlled ambient storage conditions [25 °C ± 2 °C/60% relative humidity (RH) ± 5%RH] representing tobacco flavor containing 4.0% NBW + PG/VG (30:70) + 15% H_2_O (product A) and menthol flavor containing 3.5% NBW + PG/VG (60:40) + 10% H_2_O (product B) were also used to demonstrate the method’s ability to identify the eight validation fortification compounds correctly in the presence of the complex matrix of aged e-vapor products. These e-vapor prototype products, designated as product A and product B, were only used for evaluation of selectivity during method validation and were fortified at a 10 ppm level with the mixture of validation fortification standards.

**TABLE 2:** Method assessment sample information.

Sample ID	%Base (PG:VG)	%H_2_O	%NBW
F1	82.5 (50:50)	15	2.5
F2	97.5 (50:50)	0	2.5
F3	85 (50:50)	15	0
F4	85 (80:20)	15	0
F5	85 (20:80)	15	0

### Method Validation Characteristics

The primary purpose of this method performance evaluation was to demonstrate that the method could accurately detect and identify compounds in the aerosol and e-liquid of e-vapor products. The most critical parameters for the method validation are detailed below and include accuracy, repeatability, intermediate precision, selectivity, LOD, and evaluation of false negatives. Other validation elements were evaluated but are not discussed in detail, including aerosol trapping efficiency, instrument precision, robustness, stability of sample extracts, and system suitability. The linearity as evaluated in the typical targeted analysis method validation was not evaluated as this semi-quantitative method uses a single-point calibration curve and no regression analysis was performed, including generation of coefficients of variance (*r* ^2^), as is typically done for targeted quantitative analysis techniques.

The method validation experiments were conducted using both fortified and unfortified matrix samples listed in Table 2. Samples were fortified with known amounts of the following eight compounds to evaluate method performance: hydroxyacetone, piperonal, 2,3,5-trimethylpyrazine, menthone, (E)-beta-damascone, cinnamic acid methyl ester, myosmine, and cotinine. Accuracy of this semi-quantitative method was evaluated by comparing the fortified concentrations to the measured concentrations for the eight compounds described above. Blank non-flavored e-liquid matrices listed in Table 2 (F1–F5) were fortified at three concentration levels (2 ppm, 5 ppm, and 10 ppm) with the eight compounds. Additionally, an unfortified sample for each matrix type was extracted and analyzed to identify any potential interferences to evaluate selectivity. Repeatability and intermediate precision were evaluated for each of the matrices (F1–F5) fortified at the mid-level (5 ppm) by analyzing three replicates over 3 days. The semi-quantitative concentration data for all individual replicates, mean, standard deviation, and % RSD were calculated for each day (repeatability) and over a 3 day period (intermediate precision). Selectivity was evaluated based upon the ability of the MassHunter Unknowns Analysis data processing method to detect and accurately identify compounds in fortified and unfortified samples. Peak identification included matching retention times and mass spectra from the sample to the in-house custom and the NIST mass spectral libraries. The sensitivity of the method was evaluated by determining the LOD of the method. For this, the F1 blank e-liquid matrix (Table 2) was fortified with the validation fortification standards at 0.50, 0.70, 1.0, 2.0, and 5.0 ppm. Evaluation of match factor scores and S/N threshold was used to establish the LOD. Additional validation experiments were conducted to evaluate for potential false negatives and to assess a threshold of the method to detect changes in samples to allow for differential analysis. A threshold for significant change to perform differential analysis was established to allow comparison between two samples. A limitation of the Agilent MassHunter Unknowns analysis software is that it is not possible to track the reason(s) for peak identification failure due to method parameters. For example, if any given peak is misidentified or not identified, there is no mechanism for identifying exactly which parameter(s) failed the acceptance criteria, such as match factor score, signal-to-noise ratio, or retention time window results. Also, a probability of detection curve could not be generated due to the match factor score parameters not having an impact on compound identifications at lower concentration levels. Additionally, adjustment of the S/N ratio method parameters to a lower setting caused some fortification compounds to be non-detectable (false negatives), resulting from background and instrument noise interferences, which both increased at the lower setting.

### Comprehensive Non-Targeted Analysis Profile of a Commercial Product

To demonstrate the application of the method for characterizing aerosol and e-liquid formulations from e-vapor products, we applied our workflow to perform a comprehensive chemical profile for a commercially available e-vapor product (tobacco flavor—product C), which was available at the time of analysis at local convenience stores in the Richmond, Virginia area. The analysis included e-liquid and aerosol samples at an initial time point (*T* = 0) and product aged to 6 months (*T* = 6) stored in environmental chambers under ambient storage conditions (25°C ± 2°C/60% RH ± 5% RH). The aerosol samples were generated using an intense puffing regime (a 5 s puff duration, a 55 cc puff volume, and a 30 s puff interval) for the aerosol collection and analyzed in triplicate (*n* = 3). The e-liquid samples were analyzed using an equivalent amount of e-liquid to the collected aerosol mass as the test samples and extracted with the same extraction solvent. Data processing was conducted with MassHunter Unknowns Analysis software to provide identification and quantitation of all detected peaks. All peaks identified by the software were confirmed for accuracy.

## Results and Discussion

### Accuracy

This method is a semi-quantitative method that provides estimated concentrations for the analytes detected in the samples. The estimated concentration is based upon the response factor of a single internal standard as described above. Estimation of the concentration using the response of a single internal standard is inherently less accurate than targeted methods that quantitate the concentration of an analyte using multi-level calibration curves prepared with reference standards, followed by regression analysis. In addition, the response for analytes in GC-MS with EI varies based on compound fragmentation, which is different for all compounds. To evaluate accuracy, the blank matrix samples in Table 2 (F1–F5) were fortified with the eight validation fortification standards at 2 ppm, 5 ppm, and 10 ppm. All samples were background-subtracted based upon analysis of the corresponding unfortified matrix sample (F1–F5). Accuracy was determined using the following equation:

$$\%\;Accuracy\;=\;\frac{Measured\;Amount\;\left(ppm\right)}{Background\;Amount\;\left(ppm\right)+\;Fortified\;Amount\;\left(ppm\right)}\;\times \;100\%.$$

The results of these accuracy studies are presented in Table 3. The combined average recovery for each of the levels evaluated for all analytes ranged from 82.6 to 90%. The accuracy for most matrices and fortification levels for six out of eight analytes tested fell between 70 and 120% recovery, with the exception of 2 ppm fortification in the F1 matrix for (E)-beta-damascone and the cotinine fortification in the F3 matrix, resulting in 67 and 193% accuracy, respectively. Hydroxyacetone and menthone had accuracy with values between 43 and 116%, and 47 and 52%, respectively. The results indicate that the accuracy of the method varies from approximately 43 to 193% across all analytes and matrices evaluated, Table 3. The variability for hydroxyacetone for accuracy was high in comparison to other analytes due to the inconsistency of the amount of this compound detected in the unfortified samples (see the Supplementary Material for %RSD). The lowest recovery was observed with menthone due to the difference in response factor compared to the internal standard. Cotinine’s large deviation for the F3 matrix at 2 ppm was determined to be related to matrix interferences, which resulted in issues with the peak deconvolution. In this case, the incorrect base peak was selected by the processing software, resulting in a different response for quantitation. Cotinine’s observed deviations were due to a limitation of the automated data processing software, which does not allow users to edit or change the base peak used for quantitation. These accuracy results demonstrate compound specific variability; however, data support that our NTA semi-quantitative method is fit for the intended purpose.

**TABLE 3:** Summary of accuracy results.

Matrix	Hydroxyacetone	2,3,5-Trimethyl pyrazine	Menthone	(E)-beta-damascone	Cinnamic acid methyl ester	Myosmine	Piperonal	Cotinine
% accuracy at 2 ppm								
F1	87	98	48	67	102	93	106	97
F2	116	103	50	83	107	90	116	100
F3	81	103	52	84	110	84	120	193
F4	69	99	51	78	107	79	111	99
F5	93	104	50	80	105	79	116	99
% accuracy at 5 ppm								
F1	66	108	52	83	111	94	119	108
F2	62	101	49	75	99	81	111	96
F3	63	104	50	79	107	81	116	99
F4	52	97	48	75	102	74	104	97
F5	87	108	51	79	109	77	114	102
% accuracy at 10 ppm								
F1	44	100	47	75	103	84	110	98
F2	49	102	50	77	106	81	113	99
F3	50	100	48	74	100	74	109	94
F4	43	96	48	72	98	72	105	94
F5	68	104	50	78	107	78	109	94

### Repeatability and Intermediate Precision

Repeatability, a measure of a method’s ability to generate equivalent results from multiple preparations of the same sample within a single laboratory, along with intermediate precision (over 3 days of analysis), was evaluated using the 5.0 ppm fortification level for all eight analytes in all the sample matrices listed in Table 2. A summary of the percent relative standard deviation (%RSD) for repeatability and intermediate precision, which is representative of the average of three replicates, is provided in Tables 4 and 5, respectively. For all analytes, the repeatability was between 0.2 and 14.1% with an average %RSD of 5.6%. The intermediate precision data were 22.1% RSD or lower for all eight compounds in each of the matrices. The overall average %RSD for all compounds and matrices evaluated for intermediate precision was 10.1%. The results demonstrated that the sample analysis and data processing were reproducible across multiple days and sample types.

**TABLE 4:** Repeatability results summary—%RSD (*n* = 3).

Matrix	Hydroxyacetone	2,3,5-Trimethylpyrazine	Menthone	(E)-beta-damascone	Cinnamic acid methyl ester	Myosmine	Piperonal	Cotinine
F1	7.4	7.6	9.6	6.6	6.9	5.8	9.0	4.7
F2	8.7	0.2	2.4	2.9	2.1	2.1	4.1	2.5
F3	9.4	7.5	8.4	11.7	14.1	10.6	13.5	5.3
F4	4.2	1.3	3.1	2.7	1.8	2.3	2.6	5.8
F5	1.9	3.7	6.0	3.4	6.7	3.5	8.7	2.5

**TABLE 5:** Intermediate precision summary—%RSD (*n* = 9).

Matrix	Hydroxyacetone	2,3,5-Trimethylpyrazine	Menthone	(E)-beta-damascone	Cinnamic acid methyl ester	Myosmine	Piperonal	Cotinine
F1	13.3	9.2	9.6	7.1	11.2	7.6	7.1	11.5
F2	11.2	9.5	10.1	5.6	9.5	8.8	10.2	14.5
F3	15.0	7.3	9.8	8.9	10.7	9.2	9.6	9.9
F4	8.1	7.1	9.1	10.0	9.7	8.6	9.5	11.7
F5	10.1	7.8	11.7	9.1	11.6	11.5	9.9	22.1

### Selectivity

This method is inherently selective due to the use of mass spectrometry detection. Method selectivity was evaluated using MassHunter Unknowns Analysis software based upon the ability of the processing method to detect and correctly identify compounds in aged e-vapor prototype aerosol and e-liquid samples. Product A and B samples were stored under ambient conditions (25°C ± 2°C/60%RH ± 5%RH) for approximately 2 years prior to analysis. Aerosol and e-liquid samples were fortified with the mix of eight compounds, resulting in an analyte concentration of 10 ppm of each compound. These fortified samples were analyzed in triplicate (*n* = 3) in conjunction with their corresponding unfortified samples and were treated independently during data processing. The average estimated concentrations obtained from the evaluation of these samples for fortified and unfortified samples showed an increase in the concentration for each compound in fortified samples, and 99.4% were identified correctly by MassHunter Unknowns Analysis workflow (see details in the Supplementary Material). Additionally, the frequency of correct/incorrect chemical identifications from the data processing software was evaluated for the fortified sample extracts based on the library mass spectrum match factor scores. The frequency of correct chemical identification for the fortified sample extract for each product and sample type (aerosol and e-liquid) was 99.0% (see the Supplementary Material). Thus, the experiments for method selectivity successfully demonstrated the method’s ability to perform compound identifications correctly in the presence of a complex matrix.

### Limit of Detection

The LOD of this screening method is dependent on the analyte response, and these responses must be sufficient to produce detailed mass spectra with fragmentation patterns to accurately identify compounds based on comparison of the spectra to those in the in-house and/or NIST libraries. The automated data processing by MassHunter Unknowns Analysis software generates a mass spectrum match factor score for each possible identification, which ranges between 0 and 100 (with a score of 100 being the best possible match). During the optimization of the parameters for data processing, if a peak is detected with a match factor score less than 55, then the response is insufficient to provide a reliable mass spectrum for compound identification. To determine the LOD, we evaluated the match factor score and S/N for each of the eight compounds fortified in the F1 blank e-liquid matrix samples at 0.5, 0.7, 1.0, 2.0, and 5.0 ppm. A S/N threshold of 8:1 was chosen based upon the minimum response needed for acceptable mass spectral deconvolution. We set the LOD such that at least 50% of the validation fortification compounds were identified correctly and provided a match factor score greater than 55. Out of the eight fortification compounds, the total number of compounds with confirmed identification for fortification levels of 0.5, 0.7, 1.0, 2.0, and 5.0 ppm was 3, 4, 6, 7, and 8, respectively. Samples fortified at 0.7 ppm provided acceptable identifications for four out of the eight target compounds. Piperonal, menthone, myosmine, and cotinine were correctly identified. Based on this information, the method’s LOD was determined to be 0.7 ppm.

### Evaluation of False Negatives

It is critical to evaluate the reporting limit of a screening method and understand the potential for false negative occurrences. A limit test is commonly used for the semi-quantitative screening method to set a “cutoff” or threshold value such that a false negative rate is less than 5% of the analytical results (FDA, 2019a). This is based upon the lowest concentration that would provide a response where compounds would be detected 95% of the time. Due to the complexity of e-vapor samples, it is not always feasible to evaluate the threshold value for every analyte. Therefore, we used the data from the LOD determination where 0.7 ppm was established as the lowest concentration that provided correct identification for four of the eight compounds in the F1 e-liquid matrix. The calculated concentrations for four analytes that were correctly identified in samples at the LOD provided a range of concentrations due to their differences in response factors. Myosmine and Cotinine were present in the unfortified F1 blank e-liquid samples; therefore, we conducted a blank subtraction to ensure that the threshold value was based upon the fortified concentration. After blank subtraction, the following equation was used to determine the threshold value:

Threshold Value = [Mean concentration – (t × Standard Deviation)],

where *t* = one-tailed Student’s t value for (*n*-1) degrees of freedom at the 95% confidence level. Table 6 contains the calculated concentration (ppm) for each of the four analytes used to determine the LOD with and without (corrected) blank subtraction and calculation of the threshold limit of the method.

**TABLE 6:** Calculated threshold values for fortification compounds.

Sample	Menthone	Myosmine	Piperonal	Cotinine
Blank (ppm)	NA	3.91	NA	4.97
Replicate-1 (ppm)	0.41	4.54	0.99	5.36
Replicate-1 (ppm) Corrected	0.41	0.63	0.99	0.40
Replicate-2 (ppm)	0.45	5.03	1.02	6.03
Replicate-2 (ppm) Corrected	0.45	1.12	1.02	1.07
Replicate-3 (ppm)	0.43	4.68	0.91	6.26
Replicate-3 (ppm) Corrected	0.43	0.77	0.91	1.29
Replicate-4 (ppm)	0.38	4.97	0.92	5.43
Replicate-4 (ppm) Corrected	0.38	1.06	0.92	0.46
Replicate-5 (ppm)	0.41	4.98	1.00	5.87
Replicate-5 (ppm) Corrected	0.41	1.07	1.00	0.90
Replicate-6 (ppm)	0.43	5.07	1.00	6.01
Replicate-6 (ppm) Corrected	0.43	1.16	1.00	1.05
Average (ppm)	0.42	0.969	0.974	0.86
S.D.	0.022	0.216	0.045	0.357
%RSD	5.4	22.3	4.6	41.4
Student *t*-test Value (*n*-1)	2.015	2.015	2.015	2.015
Threshold value (ppm)	0.371	0.534	0.883	0.142

The threshold value ranged from 0.142 to 0.883 ppm for these four analytes. Using the average for all analytes, we determined a threshold of 0.5 ppm. Compound concentrations above 0.5 ppm should have a sufficient response to be detected 95% of the time.

### Threshold of Significant Concentration Change

Differential analysis is a technique that allows for comparison between two samples to determine if the differences in semi-quantitative results are of statistical significance. Differential analysis is conducted by establishing a threshold of change that could be detected by the method, such as detecting increases in analyte concentration in different samples. We used a statistical approach similar to Bonferroni correction to determine the threshold for detecting differences, which takes in to account the variability associated with the replicate analysis. This was accomplished by using the data from intermediate precision F1–F5 samples fortified at 5 ppm to determine the variability observed for the estimated concentration results for each analyte. The variability attributed to intermediate precision provides an indication of what may be expected during a study and therefore represents the variability associated with the measured concentrations for different compounds.

Our approach used the method variation σ_m_ assuming an estimated variation based on 16 degrees of freedom. The value of 16 was derived from using the values for all eight identified compounds with two degrees of freedom each. The criterion | X_t_ - X_c_ | > k∙σ_m_ was used to determine whether the test product concentration (X_t_) is different from the control concentration (X_c_). The concentration of a test product that is different from the control concentration represents the calculated value (k) that is multiplied by method variation (i.e., standard deviation, S.D.).

The following equation was used to derive a reasonable value for the calculated value, k:

$$P\left(\left|{\bar{x}}_{t}-{\bar{x}}_{c}\right|>k\sigma_{m}\right)=P\left(\frac{{|\bar{x}}_{t}-{\bar{x}}_{c}|}{\sqrt{2}\sigma_{m}}>\frac{k}{\sqrt{2}}\right)\Rightarrow k=\sqrt{2}\;t$$

In the equation, *t* represents the *t*-critical value, which is the inverse of the two-tailed student’s *t*-distribution (a continuous probability distribution for testing on a small data set). In order to minimize the number of false positives, the probability (P) associated with the *t*-critical value would be inversely proportional to the number of comparisons being made. For example, assuming a scenario of 60 comparisons, the probability would be 0.05/60. In this case, *t* is equal to 4.10 and k is equal to 5.80 (rounded to 6.0 for simplicity). Therefore, a reasonable level that can be considered an increase relative to the control would be 6.0 multiplied by S.D. measured using the intermediate precision studies. Listed in Table 5 of the Supplementary Material are the determinations of fold increase used for identification of changes for all matrices. Data in the table show the days 1–3 intermediate precision means, the mean control concentration X_c_ for all intermediate precision results (*n* = 9), and the standard deviations for each of the five matrices evaluated for intermediate precision, along with the calculated value (X_t_ = mean estimated concentration + 6 S.D.) for a measurable increase. Fold increase for each compound is the ratio of test product concentration to control concentration (X_t_/X_c_). The average fold increases for F1, F2, F3, F4, and F5 are 1.37, 1.40, 1.36, 1.37, and 1.58, respectively, with an overall average of 1.42. The data from the intermediate precision were consistent; however, the data may not accurately represent the overall variation we may see over the course of a long-term stability study. Therefore, we would expect that using this data set would provide a good estimation of the minimum fold change that could be detected. Based on these data, it was determined that the method can be used to report a 1.4-fold change when comparing two samples. Individual replicate data for each analyte fortified in each matrix (F1–F5) are included in the Supplementary Material.

### Application to Commercial e-Vapor Products

To demonstrate the application of the method to characterize aerosol and e-liquid formulations from e-vapor products, we conducted comprehensive chemical profiling for a commercially available e-vapor product (tobacco flavor, 3.5% NBW—product C). The analysis included e-liquid and aerosol samples at an initial time point (*T* = 0) and product aged to 6 months (*T* = 6), stored under ambient conditions. The set of samples was analyzed in triplicate (*n* = 3), resulting in an average aerosol mass of 0.652 g for the *T* = 0 sample and 0.606 g for the *T* = 6 sample, using an intense puffing regime for the aerosol collection of over 140 puffs. The liquid samples were analyzed using an equivalent amount of the e-liquid as the average of aerosol mass for the test samples and were prepared with the same extraction solvent. Data were processed using MassHunter Unknowns Analysis to generate a list of compounds with tentative identifications. The data were then manually verified, and AMDIS software was used to confirm peak identifications and assigned identification confidence based on the NIST MS library match factor scores. Tables 7 and 8 include the results of all the compounds that were identified at *T* = 0, excluding the major ingredients in the e-vapor formulation (i.e., PG, VG, water, and nicotine), and compounds identified in the blanks. Our analysis detected 46 compounds in the e-liquid formulation and 55 compounds in the aerosols, with approximately 50% of these compounds having a confirmed identification confidence. We observed 19 peaks with an unknown identification confidence classification in the aerosol and 13 peaks in the e-liquid. Unknown compound classification was given to compounds that did not meet the acceptable match factor score criteria with the NIST library or in-house library. The process of aerosolization of the e-liquid resulted in 12 new unknown compounds, 3 of which were designated as nicotine-related compounds based on the similarity of their mass spectral fragmentation patterns to that of nicotine.

**TABLE 7:** Analysis of product C, e-liquids, and average concentration (*T* = 0, *n* = 3).

Retention time (min)	Compound	CAS#	Identification confidence	Avg (µg/gm)	Count (# of times identified)
4.08	Pyridine	110-86-1	CONFIRMED	5.29	1
4.75	Dimethoxydimethylsilane	1112-39-6	CONFIRMED	6.33	3
5.38	Ethyl lactate	97-64-3	CONFIRMED	2.85	1
6.00	Trimethylpyrazine	14667-55-1	CONFIRMED	44.80	3
6.50	Acetic acid	64-19-7	CONFIRMED	487.54	3
7.72	1-Dodecanamine, N,N-dimethyl-	112-18-5	CONFIRMED	7.28	3
7.95	Menthol	89-78-1	CONFIRMED	5.06	3
8.14	Acetylpyrazine	22047-25-2	CONFIRMED	9.36	3
8.55	2(3H)-Furanone, 5-ethyldihydro-	695-06-7	CONFIRMED	15.79	3
8.66	Unknown	0-00-0	NA	1.87	3
9.31	gamma-Heptalactone	105-21-5	CONFIRMED	20.43	3
9.36	Dipropylene glycol	110-98-5	CONFIRMED	20.01	3
9.40	beta-damascenone	23726-93-4	CONFIRMED	2.52	3
9.46	Geraniol	106-24-1	CONFIRMED	1.05	1
9.66	2-Methoxyphenol	90-05-1	CONFIRMED	46.55	3
10.11	Unknown	0-00-0	NA	0.97	3
11.08	Ethyl maltol	4940-11-8	HIGH	138.83	3
11.10	Unknown	0-00-0	NA	2.64	3
11.20	Propanoic acid, 2-hydroxy, 2-hydroxypropyl ester	14396-73-7	CONFIRMED	22.18	3
12.00	Eugenol	97-53-0	CONFIRMED	16.86	3
12.04	delta-Octalactone	698-76-0	HIGH	2.24	3
12.04	Myosmine	532-12-7	CONFIRMED	2.23	3
12.10	Unknown	0-00-0	NA	2.95	3
13.10	Beta nicotyrine	487-19-4	CONFIRMED	1.39	2
13.90	Bisabolol oxide A	22567-36-8	MEDIUM	2.25	3
14.05	Benzoic acid	65-85-0	CONFIRMED	1588.47	3
14.10	Ethanone, 1-(4-methylphenyl)-	122-00-9	CONFIRMED	2.71	1
14.60	Unknown	0-00-0	NA	1.28	2
14.75	p-Dioxane-2,5-dimethanol	14236-12-5	CONFIRMED	14.47	3
14.86	Vanillin	121-33-5	CONFIRMED	52.95	3
14.92	Bis(2,6-hydroxymethyl) dioxane	54120-69-3	CONFIRMED	51.93	3
15.14	Bis(2,6-hydroxymethyl) dioxane—Iso2	54120-69-3	HIGH	6.42	2
15.15	Bis(2,6-hydroxymethyl) dioxane—Iso3	54120-69-3	HIGH	24.60	3
15.19	Unknown	0-00-0	NA	1.80	1
15.26	Bis(2,6-hydroxymethyl) dioxane—Iso4	54120-69-3	HIGH	35.78	3
15.30	Guaiacyl acetone	2503-46-0	CONFIRMED	1.16	3
15.40	Bis(2,6-hydroxymethyl) dioxane—Iso5	54120-69-3	HIGH	10.47	3
16.29	Unknown long-chain alkane	0-00-0	NA	1.13	1
16.70	Cotinine	486-56-6	CONFIRMED	2.18	3
16.96	Unknown long-chain alkane	0-00-0	NA	4.15	1
17.60	Unknown long-chain alkane	0-00-0	NA	6.67	1
18.64	Unknown long-chain alkane	0-00-0	NA	8.48	1
19.58	Unknown long-chain alkane	0-00-0	NA	10.33	1
22.45	Unknown	0-00-0	NA	7.83	1
22.46	Unknown	0-00-0	NA	6.62	1

NA is not applicable; Iso—Isomer.

**TABLE 8:** Analysis of product C, aerosols, and average concentration (*T* = 0, *n* = 3).

Retention time (min)	Compound	CAS#	Identification confidence	Avg (µg/gm)	Count (# of times identified)
3.72	Decamethylcyclopentasiloxane	541-02-6	CONFIRMED	13.48	3
4.75	Dimethoxydimethylsilane	1112-39-6	CONFIRMED	5.45	3
5.04	Hydroxyacetone	116-09-6	CONFIRMED	8.59	3
5.28	Dodecamethylcyclohexasiloxane	540-97-6	CONFIRMED	8.98	3
6.00	Trimethylpyrazine	14667-55-1	CONFIRMED	37.41	3
6.50	Acetic acid	64-19-7	CONFIRMED	446.47	3
6.80	Tetradecamethylcycloheptasiloxane	107-50-6	CONFIRMED	2.48	3
7.72	1-Dodecanamine, N,N-dimethyl-	112-18-5	CONFIRMED	5.12	1
7.95	Menthol	89-78-1	CONFIRMED	5.20	3
8.14	Acetylpyrazine	22047-25-2	CONFIRMED	8.10	3
8.31	Hexadecamethylcyclooctasiloxane	556-68-3	HIGH	0.84	3
8.55	2(3H)-Furanone, 5-ethyldihydro-	695-06-7	CONFIRMED	14.96	3
8.66	Unknown	0-00-0	NA	3.85	3
9.13	Unknown	0-00-0	NA	0.74	1
9.16	N,N-Dimethyltetradecanamine	112-75-4	CONFIRMED	4.22	1
9.31	gamma-Heptalactone	105-21-5	CONFIRMED	19.94	3
9.36	Dipropylene glycol	110-98-5	CONFIRMED	19.58	3
9.40	beta-Damascenone	23726-93-4	CONFIRMED	3.08	3
9.46	Geraniol	106-24-1	CONFIRMED	0.94	2
9.66	2-Methoxyphenol	90-05-1	CONFIRMED	41.72	3
10.11	Unknown	0-00-0	NA	0.94	3
11.08	Ethyl maltol	4940-11-8	HIGH	129.63	3
11.10	Unknown	0-00-0	NA	1.53	3
11.20	Propanoic acid, 2-hydroxy, 2-hydroxypropyl ester	14396-73-7	CONFIRMED	27.92	3
11.30	Unknown	0-00-0	NA	0.80	1
12.00	Eugenol	97-53-0	CONFIRMED	15.42	3
12.04	delta-Octalactone	698-76-0	HIGH	3.34	3
12.04	Myosmine	532-12-7	CONFIRMED	2.83	3
12.10	Unknown	0-00-0	NA	3.08	3
12.30	Unknown	0-00-0	NA	0.82	1
12.70	Unknown nicotine-related compound	0-00-0	NA	0.87	3
13.10	Beta nicotyrine	487-19-4	CONFIRMED	4.09	3
13.90	Bisabolol oxide A	22567-36-8	MEDIUM	2.16	3
13.90	Unknown nicotine-related compound	0-00-0	NA	1.41	3
14.05	Benzoic acid	65-85-0	CONFIRMED	1617.05	3
14.40	Unknown	0-00-0	NA	0.75	2
14.50	Unknown	0-00-0	NA	2.51	3
14.60	Unknown	0-00-0	NA	1.27	2
14.75	p-Dioxane-2,5-dimethanol	14236-12-5	CONFIRMED	15.52	3
14.86	Vanillin	121-33-5	CONFIRMED	51.70	3
14.87	1-Octadecanol	112-92-5	CONFIRMED	0.75	2
14.92	Bis(2,6-hydroxymethyl) dioxane	54120-69-3	CONFIRMED	58.49	3
15.14	Bis(2,6-hydroxymethyl) dioxane—Iso2	54120-69-3	HIGH	12.21	1
15.15	Bis(2,6-hydroxymethyl) dioxane—Iso3	54120-69-3	HIGH	24.98	3
15.20	Unknown	0-00-0	NA	2.13	3
15.26	Bis(2,6-hydroxymethyl) dioxane—Iso4	54120-69-3	HIGH	37.37	3
15.30	Guaiacyl acetone	2503-46-0	CONFIRMED	1.47	3
15.40	Bis(2,6-hydroxymethyl) dioxane—Iso5	54120-69-3	HIGH	11.51	3
16.70	Cotinine	486-56-6	CONFIRMED	2.76	3
17.00	Unknown nicotine-related compound	0-00-0	NA	1.34	2
17.60	Unknown long-chain alkane	0-00-0	NA	2.48	1
18.50	Unknown	0-00-0	NA	1.02	2
18.60	Unknown	0-00-0	NA	2.10	2
18.70	Unknown	0-00-0	NA	0.77	1
19.58	Unknown long-chain alkane	0-00-0	NA	1.60	1

NA is not applicable; Iso—Isomer.

Differential analysis between the *T* = 0 and *T* = 6 samples was conducted for both aerosol and e-liquid samples. There were 14 additional compounds in the e-liquid and 19 additional compounds detected in the aerosol generated from the aged sample compared to the corresponding initial (*T* = 0) profiles of the e-liquid and aerosol (see the Supplementary Material for details). These additional compounds found at the *T* = 6 time point included various chemical classes related to flavors (e.g., beta-citronellol, delta-decalactone, ethanone,1(-3-pyridinyl), hexanal, etc.), nicotine degradation products (e.g., n-methylnicotinamide, 3,4-dipyridyl ketone), leachable compounds (e.g., a siloxane, diethoxydimethylsilane), and nine unidentified compounds. The tentatively identified compounds that were not confirmed with a reference standard and unknown compounds would require additional characterization of the peaks, such as HRMS, NMR, and expert evaluation, for structure elucidation. The additional peak characterization was outside of the scope of this article.

## Conclusions

As demonstrated by our chemical characterization, e-vapor products are a complex mixture that contains a variety of chemicals including flavor-related compounds in addition to the typical primary formulation ingredients PG, VG, and nicotine. We have provided a novel non-targeted analysis approach for chemical characterization of aerosols and e-liquids in e-vapor products using an automated data processing workflow. The GC-MS profiling method performance was validated, and criteria were established for precision, accuracy, selectivity, and LOD. In addition, other unique validation elements deemed necessary for an NTA method, such as evaluating potential for occurrence of false negatives and threshold of significant concentration change, were evaluated. MassHunter Unknowns Analysis method parameters were optimized to ensure the method’s ability to perform the automated peak picking, deconvolution, and compound identifications with an appropriate match factor from the available library and provide semi-quantitative concentration for each compound. The validation parameters of precision and accuracy had a %RSD of less than or equal to 8.5% for all matrices and concentration levels. Estimated concentrations ranged from 0.5 to 2 times the actual value, as calculated based on the manual response factor of the internal standard. This method was able to detect a 1.4-fold change in a compound level when comparing two samples. The LOD of this method was determined to be 0.7 ppm. In the absence of guidance documents for validation of non-targeted methods, the semi-quantitative NTA method validation described here is an example of potential best practices and was successful in determining the method to be fit for the purpose of comprehensive screening of e-vapor products. Evaluation of the commercial e-vapor, product e-liquid, and aerosol demonstrates the ability of the automated data processing method to identify compounds consistently across time and to detect new compounds that may form during aging. Overall, this approach is applicable for the chemical characterization of volatile and semi-volatile compounds in the e-liquids and aerosols of e-vapor products to support the assessment of the products, including toxicological risk assessments, for the FDA’s PMTA authorization pathway.

## Data Availability

The original contribution presented in the study is included in the article/Supplementary Material, and further inquiries can be directed to the corresponding author.
