# Supplementary material for: Non-Targeted Analysis Using Gas Chromatography-Mass Spectrometry for Evaluation of Chemical Composition of E-Vapor Products
Source: Front Chem. 2021 Sep 29;9:742854. doi: 10.3389/fchem.2021.742854 (PMC8511636; doi:10.3389/fchem.2021.742854)
Supplement: Supplementary file 1 [file DataSheet1.pdf]

Supplementary Material

Non-Targeted Analysis Using Gas Chromatography Mass Spectrometry for Evaluation of Chemical Composition of E-Vapor Products

Niti H. Shah*, Michael R. Noe, Kimberly A. Agnew-Heard, Yezdi B. Pithawalla, William P. Gardner, Saibal Chakraborty, Nicholas McCutcheon, Hannah Grisevich, Thomas J. Hurst, Michael J. Morton, Matt S. Melvin, John H. Miller IV

Center for Research & Technology, Altria Client Services LLC, Richmond, VA, United States

*Corresponding author: Niti.H.Shah@altria.com; Phone: 804-335-2604; Fax: 804-335-2087

Accuracy

A summary of the percent relative standard deviation (%RSD) accuracy results for the 2ppm, 5ppm, and 10ppm fortified matrices are presented for each analyte in Table 1 below.

Table 1. Summary of accuracy results: 2ppm, 5ppm and 10 ppm

%RSD at 2PPM	Hydroxyacetone	2,3,5-trimethyl pyrazine	Menthone	(E)-Beta-Damascone	Cinnamic acid methyl ester	Myosmine	Piperonal	Cotinine
F1	NA*	21.08	18.81	19.15	23.17	26.95	24.33	22.72
F2	1.78	6.47	4.87	1.57	2.01	0.43	1.88	3.00
F3	9.05	9.55	7.50	6.74	6.33	7.75	7.12	78.89**
F4	4.98	1.12	3.34	2.24	3.69	4.37	2.77	7.13
F5	10.52	7.12	8.27	4.60	11.52	7.77	6.35	8.88
%RSD at 5PPM	Hydroxyacetone	2,3,5-trimethylpyrazine	Menthone	(E)-Beta-Damascone	Cinnamic acid methyl ester	Myosmine	Piperonal	Cotinine
F1	7.43	7.58	9.56	6.60	6.91	5.83	9.03	4.70
F2	8.68	0.17	2.42	2.87	2.08	2.10	4.12	2.47
F3	9.41	7.55	8.40	11.68	14.12	10.61	13.54	5.30
F4	4.19	1.26	3.07	2.73	1.80	2.31	2.57	5.84
F5	1.91	3.74	6.01	3.36	6.66	3.52	8.67	2.51
%RSD at 10PPM	Hydroxyacetone	2,3,5-trimethyl pyrazine	Menthone	(E)-Beta-Damascone	Cinnamic acid methyl ester	Myosmine	Piperonal	Cotinine
F1	9.90	2.78	2.26	0.40	1.66	1.90	1.83	1.69
F2	1.85	1.23	3.12	2.47	0.10	1.02	1.77	3.06
F3	3.48	0.84	4.44	2.16	1.42	1.34	3.79	1.83
F4	5.59	4.31	3.32	4.67	4.36	2.38	4.46	6.39
F5	3.35	2.47	4.27	1.67	2.82	3.97	5.52	3.22

* %RSD could not be calculated because two of the three replicates were below LOD/not detected.
** Highest or lowest of the result set.

Selectivity

Summary of selectivity data (Table 2 and Table 3) for Product A and Product B e-vapor prototype products that were aged ~2 years.

Table 2. Product A and Product B e-liquid and aerosol fortification results.

Compound	Product A – Liquid*	Product B – Liquid*	Product A – Aerosol*	Product B – Aerosol*
Hydroxyacetone Unfortified (PPM)	ND	10.97	23.26	14.88
Hydroxyacetone Fortified (PPM)	6.22	13.85	29.07	21.15
**Difference (PPM)**	**6.22**	**2.89**	**5.80**	**6.28**
Trimethylpyrazine Unfortified (PPM)	ND	ND	ND	ND
Trimethylpyrazine Fortified (PPM)	16.09	13.97	24.37	21.51
**Difference (PPM)**	**16.09**	**13.97**	**24.37**	**21.51**
Menthone Unfortified (PPM)	ND	304.63	ND	266.63
Menthone Fortified (PPM)	7.20	309.86	11.62	290.88
**Difference (PPM)**	**7.20**	**5.23**	**11.62**	**24.25**
(E)-Beta-Damascone Unfortified (PPM)	ND	ND	ND	ND
(E)-Beta-Damascone Fortified (PPM)	12.37	20.74	18.38	21.68
**Difference (PPM)**	**12.37**	**20.74**	**18.38**	**21.68**
Cinnamic acid methyl ester Unfortified (PPM)	ND	ND	ND	ND
Cinnamic acid methyl ester Fortified (PPM)	15.44	14.92	23.84	21.84
**Difference (PPM)**	**15.44**	**14.92**	**23.84**	**21.84**
Myosmine Unfortified (PPM)	7.08	16.48	10.85	22.01
Myosmine Fortified (PPM)	17.41	28.50	27.35	37.16
**Difference (PPM)**	**10.33**	**12.02**	**16.49**	**15.15**
Piperonal Unfortified (PPM)	ND	ND	ND	ND
Piperonal Fortified (PPM)	16.58	17.12	24.32	23.12
**Difference (PPM)**	**16.58**	**17.12**	**24.32**	**23.12**
Cotinine Unfortified (PPM)	4.88	4.53	8.68	8.50
Cotinine Fortified (PPM)	16.25	17.17	24.80	24.21
**Difference (PPM)**	**11.37**	**12.64**	**16.12**	**15.70**

(* The average estimated concentration results for 3 replicates, n=3.)

Table 3. Product A and Product B e-liquid and aerosol average match factor scores and correct identification

Selectivity samples	Average Match Factor Score	Correct/Incorrect	% Identified correctly
Product A Unfortified (E-liquid)	95.0	NA	NA
Product A Fortified (E-liquid)	90.1	23/24	95.8
Product B Unfortified (E-liquid)	94.6	NA	NA
Product B Fortified (E-liquid)	91.6	24/24	100.0
Product A Unfortified (Aerosol)	92.6	NA	NA
Product A Fortified (Aerosol)	92.8	24/24	100.0
Product B Unfortified (Aerosol)	92.4	NA	NA
Product B Fortified (Aerosol)	91.6	24/24	100.0
Overall Average	92.6	95/96	99.0

Limit of Detection

Summary of the match factor socre and S/N for each analyte for 0.7 ppm fortified F1-matrix analysis.

Table 4: Analysis of 0.7ppm fortified F1 matrix

F1 Matrix	Compound	PPM (Estimated Conc)*	Match Factor	S/N
Blank	Hydroxyacetone	ND	NA	NA
Replicate 1		ND	NA	NA
Replicate 2		ND	NA	NA
Replicate 3		ND	NA	NA
Replicate 4		ND	NA	NA
Replicate 5		ND	NA	NA
Replicate 6		ND	NA	NA
Blank	Trimethylpyrazine	ND	NA	NA
Replicate 1		ND	NA	NA
Replicate 2		ND	NA	NA
Replicate 3		ND	NA	NA
Replicate 4		ND	NA	NA
Replicate 5		ND	NA	NA
Replicate 6		ND	NA	NA
Blank	Menthone	ND	NA	NA
Replicate 1		0.41	78.8	17.61
Replicate 2		0.45	89.6	18.66
Replicate 3		0.43	78.7	15.54
Replicate 4		0.38	78.8	16.31
Replicate 5		0.41	78.7	19.27
Replicate 6		0.43	78.8	17.93
Blank	(E)-Beta-Damascone	ND	NA	NA
Replicate 1		ND	NA	NA

F1 Matrix	Compound	PPM (Estimated Conc)*	Match Factor	S/N
Replicate 2		ND	NA	NA
Replicate 3		ND	NA	NA
Replicate 4		ND	NA	NA
Replicate 5		ND	NA	NA
Replicate 6		ND	NA	NA
Blank	Cinnamic acid methyl ester	ND	NA	NA
Replicate 1		0.97	88	8.59
Replicate 2		0.89	88	8.45
Replicate 3		ND	NA	NA
Replicate 4		ND	NA	NA
Replicate 5		ND	NA	NA
Replicate 6		ND	NA	NA
Blank	Myosmine	3.91	93.9	81.09
Replicate 1		4.54	93.9	94.1
Replicate 2		5.03	92.9	118.79
Replicate 3		4.68	93.3	69.84
Replicate 4		4.97	92.2	100.4
Replicate 5		4.98	92.4	97.06
Replicate 6		5.07	92.2	106.52
Blank	Piperonal	ND	NA	NA
Replicate 1		0.99	71.9	45.8
Replicate 2		1.02	71.9	29.96
Replicate 3		0.91	72	30.5
Replicate 4		0.92	71.9	27.79
Replicate 5		1.00	71.9	38.99
Replicate 6		1.00	72	35.02
Blank	Cotinine	4.97	92.4	121.71
Replicate 1		5.36	93.2	131.85
Replicate 2		6.03	94.2	145.55
Replicate 3		6.26	92.8	132.91
Replicate 4		5.43	86.8	113.54
Replicate 5		5.87	92.8	97.43
Replicate 6		6.01	91.6	126.7

(ND - Not detected; NA - Not applicable)

Threshold of significant change

Individual replicate data for determination of threshold for significant change for all matrices is summarized in Table 5. Fold increase was calculated as (Xt) devided by (Xc), where, Xc is the grand mean of the three day intermediate precision value for each analyte and Xt is the calcualted value for a measurable increase, (Xc+6S.D.).

Table 5. Determination of fold increase used for identification of changes for all matrices

	Hydroxyacetone	2,3,5-trimethylpyrazine	Menthone	(E)-Beta-Damascone	Cinnamic acid methyl ester	Myosmine	Piperonal	Cotinine
				F1				
Day 1 Mean	3.10	5.45	2.60	4.32	5.55	7.63	6.24	9.45
Day 2 Mean	2.49	4.74	2.37	ND	5.04	7.38	5.91	8.11
Day 3 Mean	3.27	5.08	2.44	4.17	5.00	8.15	6.19	9.20
x_c	2.95	5.09	2.47	4.24	5.19	7.72	6.11	8.92
S.D.	0.41	0.35	0.12	0.10	0.31	0.39	0.18	0.71
x_t	5.42	7.21	3.17	4.85	7.04	10.09	7.19	13.20
Fold Increase	1.84	1.42	1.28	1.14	1.35	1.31	1.18	1.48
				F2				
Day 1 Mean	2.91	5.06	2.47	3.94	4.98	6.41	5.78	8.80
Day 2 Mean	3.13	5.36	2.71	ND	5.69	7.41	6.60	8.83
Day 3 Mean	3.51	5.19	2.63	4.23	5.34	6.98	5.74	7.38
x_c	3.19	5.20	2.60	4.08	5.34	6.93	6.04	8.33
S.D.	0.30	0.15	0.12	0.20	0.36	0.50	0.49	0.83
x_t	5.01	6.11	3.31	5.31	7.47	9.93	8.96	13.29
Fold Increase	1.57	1.17	1.27	1.30	1.40	1.43	1.48	1.59
				F3				
Day 1 Mean	5.50	5.23	2.53	4.13	5.37	4.11	6.07	4.84
Day 2 Mean	4.29	5.08	2.51	ND	5.27	4.23	6.25	4.60
Day 3 Mean	6.09	5.48	2.79	4.38	5.66	4.28	6.35	5.44
x_c	5.29	5.26	2.61	4.26	5.43	4.20	6.22	4.96
S.D.	0.92	0.20	0.16	0.18	0.20	0.08	0.14	0.43
x_t	10.81	6.46	3.54	5.32	6.64	4.71	7.06	7.53
Fold Increase	2.04	1.23	1.36	1.25	1.22	1.12	1.13	1.52
				F4				
Day 1 Mean	4.26	4.90	2.40	3.90	5.12	3.76	5.44	4.79
Day 2 Mean	4.21	4.86	2.48	ND	5.19	4.07	5.91	4.34
Day 3 Mean	4.66	5.32	2.76	4.27	5.19	4.23	6.20	5.52
x_c	4.38	5.03	2.55	4.09	5.16	4.02	5.85	4.88
S.D.	0.25	0.25	0.19	0.26	0.04	0.24	0.38	0.60
x_t	5.88	6.55	3.66	5.64	5.41	5.44	8.15	8.47
Fold Increase	1.34	1.30	1.44	1.38	1.05	1.35	1.39	1.74
				F5				
Day 1 Mean	13.86	5.41	2.55	4.10	5.47	3.96	5.97	5.02
Day 2 Mean	11.47	5.45	2.70	ND	5.77	4.44	6.66	4.66
Day 3 Mean	13.49	6.04	2.81	4.69	6.13	4.95	6.76	7.03
x_c	12.94	5.63	2.69	4.40	5.79	4.45	6.46	5.57
S.D.	1.28	0.35	0.13	0.42	0.33	0.50	0.43	1.28
x_t	20.64	7.74	3.46	6.89	7.79	7.44	9.04	13.23

Fold Increase	1.60	1.37	1.29	1.57	1.35	1.67	1.40	2.38

MassHunter Unknowns Analysis parameters for data processing method

Following parameters were optimized specifically for NTA method workflow discussed in the manuscript. Peak detection: “Deconvolution”, Peak filter – exclude m/z 28, S/N – 6, Absolute area ≥ 8000 counts, Absolute height ≥ 1000 counts; Deconvolution: RT window size factor – 45, Left and Right m/z Δ = 0.5, m/z Δ units – AMU, Do not select “Use integer m/z values”. Component shape: Sharpness Threshold = 25%; Ion peaks: Min number of ion peaks – 1, Max number of ion peak shapes to store: 6; Library search: Select all relevant libraries, Pre-search type – Normal, Match factor (only applicable to custom library) – Use RT match, RT penalty – Trapezoidal, RT range – 20 sec, Penalty free range 18 sec, mismatch penalty – Additive, Max RT penalty 20; Compound Identification: Max Hit count - 1, Min match factor - 55, Min m/z - 30, Library search type - Spectral search, Multi-Library Search type - Stop when found;

Target match: Select – Qualifier ion ratios, Hit ion - Target ion and qualifier ion(s), Hit RT – Within target RT window, Additional target hit match – select “Use compound name”; Estimation response factor (RF): Select Manual RF, Manual response factor – Enter the calculate manual response factor; Blank subtraction: Perform blank subtraction – de-select, Retention time window – FWHM – “5” times, Peak threshold – Select component area, 10%.

Table 6: Analysis of commercial product 3.5% NBW (T=6), e-liquid, Average Conc.(n=3)

Retention time (min)	Compound	CAS#	Identification Confidence	Avg (µg/gm)	Count (# of Times Identified)
3.33	Hexanal*	66-25-1	HIGH	1.62	3
4.08	Pyridine	110-86-1	CONFIRMED	5.80	3
4.75	Dimethoxydimethylsilane*	1112-39-6	CONFIRMED	28.86	3
5.04	Hydroxyacetone*	116-09-6	CONFIRMED	13.28	3
5.61	1,4-diethyl-benzene*	105-05-5	MEDIUM	1.49	3
6.00	Trimethylpyrazine	14667-55-1	CONFIRMED	47.50	3
6.50	Acetic acid	64-19-7	CONFIRMED	588.60	3
7.72	1-Dodecanamine, N,N-dimethyl-	112-18-5	CONFIRMED	12.78	3
7.95	Menthol	89-78-1	CONFIRMED	5.30	3
8.14	Acetylpyrazine	22047-25-2	CONFIRMED	7.85	3
8.45	Diethoxydimethylsilane	78-62-6	CONFIRMED	25.46	3
8.50	Unknown*	0-00-0	NA	3.77	3
8.55	2(3H)-Furanone, 5-ethyldihydro-	695-06-7	CONFIRMED	15.06	3
8.66	Unknown	0-00-0	NA	2.25	3
8.88	.beta.-Citronellol*	106-22-9	CONFIRMED	0.74	2
9.16	N,N-Dimethyltetradecanamine*	112-75-4	CONFIRMED	6.24	2
9.31	gamma-Heptalactone	105-21-5	CONFIRMED	18.95	3
9.36	Dipropylene glycol	110-98-5	CONFIRMED	24.29	3
9.40	beta-Damascenone	23726-93-4	CONFIRMED	1.71	3
9.50	Ethanone, 1-(3-pyridinyl)-*	350-03-8	CONFIRMED	1.02	3
9.66	2-Methoxyphenol	90-05-1	CONFIRMED	43.27	3
10.11	Unknown	0-00-0	NA	1.11	3
11.08	Ethyl Maltol	4940-11-8	HIGH	124.89	3
11.50	Unknown*	0-00-0	NA	1.31	1
12.00	Eugenol	97-53-0	CONFIRMED	5.24	3
12.04	Myosmine	532-12-7	CONFIRMED	4.25	3
12.38	delta-Decalactone*	705-86-2	CONFIRMED	4.04	3
13.10	beta nicotyrine	487-19-4	CONFIRMED	10.54	3
13.90	Bisabolol oxide A	22567-36-8	MEDIUM	2.12	3
14.05	Benzoic Acid	65-85-0	CONFIRMED	1896.10	1
14.60	Unknown	0-00-0	NA	1.98	2
14.70	Unknown*	0-00-0	NA	24.78	3
14.75	p-Dioxane-2,5-dimethanol	14236-12-5	CONFIRMED	12.23	3
14.86	Vanillin	121-33-5	CONFIRMED	44.01	3
14.92	Bis(2,6-hydroxymethyl) dioxane	54120-69-3	CONFIRMED	21.40	3
15.14	Bis(2,6-hydroxymethyl) dioxane - Iso2	0-00-0	HIGH	3.28	1
15.15	Bis(2,6-hydroxymethyl) dioxane - Iso3	0-00-0	HIGH	27.92	3
15.26	Bis(2,6-hydroxymethyl) dioxane - Iso4	0-00-0	HIGH	8.84	3

Retention time (min)	Compound	CAS#	Identification Confidence	Avg (µg/gm)	Count (# of Times Identified)
15.30	Guaiacyl acetone	2503-46-0	CONFIRMED	7.24	3
15.40	Bis(2,6-hydroxymethyl) dioxane - Iso5	0-00-0	HIGH	46.05	3
15.88	N-Methylnicotinamide*	114-33-0	CONFIRMED	0.86	3
16.30	3,4-Dipyridyl Ketone*	0-00-0	CONFIRMED	1.11	3
16.70	Cotinine	486-56-6	CONFIRMED	3.19	3
16.96	Unknown Long Chain Alkane	0-00-0	NA	3.80	2
17.60	Unknown Long Chain Alkane	0-00-0	NA	4.74	2
18.64	Unknown Long Chain Alkane	0-00-0	NA	6.07	2
19.58	Unknown Long Chain Alkane	0-00-0	NA	6.69	2
21.19	Unknown Long Chain Alkane*	0-00-0	NA	3.17	1

NA is not applicable; * New compounds at T=6

Table 7: Analysis of commercial product 3.5% NBW (T=6), aerosol, Average Conc.(n=3)

Retention time (min)	Compound	CAS#	Identification Confidence	Avg (µg/gm)	Count (# of Times Identified)
3.33	Hexanal*	66-25-1	HIGH	1.21	2
3.72	Decamethylcyclopentasiloxane	541-02-6	CONFIRMED	15.03	3
4.08	Pyridine*	110-86-1	CONFIRMED	9.97	3
5.04	Hydroxyacetone	116-09-6	CONFIRMED	25.38	3
5.61	1,4-diethyl-benzene*	105-05-5	MEDIUM	0.79	3
6.00	Trimethylpyrazine	14667-55-1	CONFIRMED	36.46	3
6.50	Acetic acid	64-19-7	CONFIRMED	566.17	3
7.95	Menthol	89-78-1	CONFIRMED	6.72	3
8.14	Acetylpyrazine	22047-25-2	CONFIRMED	6.26	3
8.45	Diethoxydimethylsilane*	78-62-6	CONFIRMED	18.30	3
8.55	2(3H)-Furanone, 5-ethyldihydro-	695-06-7	CONFIRMED	14.37	3
8.66	Unknown	0-00-0	NA	2.20	3
9.16	N,N-Dimethyltetradecanamine	112-75-4	CONFIRMED	6.14	1
9.31	gamma-Heptalactone	105-21-5	CONFIRMED	19.38	3
9.36	Dipropylene glycol	110-98-5	CONFIRMED	23.58	3
9.40	beta-Damascenone	23726-93-4	CONFIRMED	2.28	3
9.50	Ethanone, 1-(3-pyridinyl)-*	350-03-8	CONFIRMED	0.92	3
9.66	2-Methoxyphenol	90-05-1	CONFIRMED	38.30	3
10.10	Phenethyl alcohol*	60-12-8	CONFIRMED	0.97	2
10.11	Unknown	0-00-0	NA	0.97	3
10.55	Phenol*	108-95-2	CONFIRMED	2.05	2
11.08	Ethyl Maltol	4940-11-8	HIGH	111.81	3
12.00	Eugenol	97-53-0	CONFIRMED	4.36	3

Retention time (min)	Compound	CAS#	Identification Confidence	Avg (µg/gm)	Count (# of Times Identified)
12.04	Myosmine	532-12-7	CONFIRMED	5.40	3
12.38	delta-Decalactone*	705-86-2	CONFIRMED	5.40	3
12.70	Unknown Nicotine Releated Compound	0-00-0	NA	2.10	3
13.10	beta nicotyrine	487-19-4	CONFIRMED	14.32	3
13.90	Bisabolol oxide A	22567-36-8	MEDIUM	2.01	3
13.90	Unknown Nicotine Releated Compound	0-00-0	NA	3.38	3
14.05	Benzoic Acid	65-85-0	CONFIRMED	1811.81	3
14.75	p-Dioxane-2,5-dimethanol	14236-12-5	CONFIRMED	17.27	3
14.86	Vanillin	121-33-5	CONFIRMED	45.24	3
14.92	Bis(2,6-hydroxymethyl) dioxane	54120-69-3	CONFIRMED	31.36	3
15.01	Unknown*	0-00-0	NA	26.69	3
15.14	Bis(2,6-hydroxymethyl) dioxane - Iso2	0-00-0	HIGH	4.08	3
15.15	Bis(2,6-hydroxymethyl) dioxane - Iso3	0-00-0	HIGH	40.64	3
15.20	Unknown	0-00-0	NA	2.41	2
15.26	Bis(2,6-hydroxymethyl) dioxane - Iso4	0-00-0	HIGH	12.12	3
15.30	Guaiacyl acetone	2503-46-0	CONFIRMED	5.52	3
15.40	Bis(2,6-hydroxymethyl) dioxane - Iso5	0-00-0	HIGH	70.15	3
15.88	N-Methylnicotinamide*	114-33-0	CONFIRMED	1.46	3
16.30	3,4-Dipyridyl Ketone*	0-00-0	CONFIRMED	1.86	3
16.70	Cotinine	486-56-6	CONFIRMED	4.89	3
16.80	Hexadecanoic Acid*	57-10-3	CONFIRMED	11.83	3
16.96	Unknown Long Chain Alkane*	0-00-0	NA	7.57	1
17.00	Unknown Nicotine Releated Compound	0-00-0	NA	1.71	3
17.60	Unknown Long Chain Alkane	0-00-0	NA	6.88	2
18.64	Unknown Long Chain Alkane*	0-00-0	NA	14.18	1
18.94	Octadecanoic acid*	57-11-4	CONFIRMED	9.70	2
19.48	Unknown*	0-00-0	NA	1.78	1
19.58	Unknown Long Chain Alkane	0-00-0	NA	17.36	1
20.13	Unknown Nicotine Releated Compound*	0-00-0	NA	3.68	3
20.22	Unknown*	0-00-0	NA	1.93	3
21.19	Unknown Long Chain Alkane*	0-00-0	NA	14.41	1

NA is not applicable; * New compounds at T=6
